# Evaluating Pharmacists’ Knowledge of Food–Drug Interactions in Croatia: Identifying Gaps and Opportunities

**DOI:** 10.3390/pharmacy12060172

**Published:** 2024-11-21

**Authors:** Josipa Bukić, Doris Rušić, Antonela Turic, Dario Leskur, Toni Durdov, Joško Božić, Martin Kondža, Darko Modun, Ana Šešelja Perišin

**Affiliations:** 1 Department of Pharmacy, University of Split School of Medicine, 21000 Split, Croatia; jbukic@mefst.hr (J.B.); drusic@mefst.hr (D.R.); antonela.turic.1@gmail.com (A.T.); dleskur@mefst.hr (D.L.); toni.durdov@mefst.hr (T.D.); aperisin@mefst.hr (A.Š.P.); 2 Department of Laboratory Medicine and Pharmacy, Faculty of Medicine, Josip Juraj Strossmayer University of Osijek, 31000 Osijek, Croatia; 3 Department of Pathophysiology, University of Split School of Medicine, 21000 Split, Croatia; jbozic@mefst.hr; 4 Faculty of Pharmacy, University of Mostar, 88000 Mostar, Bosnia and Herzegovina; martin.kondza@farf.sum.ba; 5 Faculty of Food and Technology, Josip Juraj Strossmayer University of Osijek, 31000 Osijek, Croatia

**Keywords:** food–drug interactions, knowledge, pharmacists, bioavailability, pharmacokinetics, food, diet, education

## Abstract

Food–drug interactions (FDIs) are pharmacokinetic or pharmacodynamic changes in drug effects caused by the presence of specific foods. To identify and prevent FDIs, pharmacists, alongside other healthcare professionals, should possess a certain level of knowledge. This study aimed to assess knowledge of FDIs among Croatian pharmacists. A total of 206 participants were included in this cross-sectional study. The median knowledge score among Croatian pharmacists was 69.44%, with an interquartile range of 19.44. Croatian pharmacists most commonly recognized FDIs involving theophylline, warfarin, and tetracycline, while the lowest rate of correct answers was observed with digoxin interactions. Future studies should evaluate pharmacists’ clinical practice concerning FDIs. Additionally, more research is needed to develop educational programs on this topic, either at the university level or for continuing education.

## 1. Introduction

Globally, drug consumption continues to increase, and patients are commonly taking multiple drugs for their conditions. This practice, polypharmacy, is frequently associated with more negative outcomes, often due to interactions, which include food–drug interactions (FDIs). Relative to other types of interactions, FDIs are not as heavily focused upon despite their potential to cause problems [1,2,3]. In terms of FDIs, it is not important whether the food patients consume is healthy or not as even diets deemed the healthiest, such as the Mediterranean diet, have significant potential for developing FDIs [4]. Understanding and controlling FDIs could lead to a reduced number of adverse drug events (ADEs) [5].

Numerous FDIs have been described in the literature, and their occurrence across different countries ranges from 6% to 70% [6]. Some well-known FDIs are interactions between tyramine-rich food and MAO inhibitors, grapefruit juice, and drugs metabolized through CYP3A4 and dairy products with tetracycline antibiotics [7,8]. The importance of FDIs and adhering to the recommended therapy regimen is very well demonstrated in the example of levothyroxine. If not taken 30 min, or even 60 min as newer studies found, before the meal, levothyroxine’s efficiency is decreased significantly [9]. One of the most investigated drugs regarding its interactions is warfarin. Warfarin has major interactions with 14 herbs or dietary supplements and moderate interaction when taken with 38 of them. Moreover, alcohol interferes with warfarin’s liver metabolism and also displaces it from plasma proteins, which results in an increased coagulant effect. The most described warfarin interactions are those with vitamin K-rich vegetables, grapefruit juice, the ginkgo biloba, etc. [10].

The role of healthcare professionals (HCPs) is not only to educate patients about FDIs but also to anticipate, recognize, and resolve FDIs when they occur in practice [11]. Since community pharmacists regularly interact with patients regarding their pharmacotherapy, they have the highest potential and probability to recognize FDIs [12]. A previous study reported that most pharmacists did not have any formal education about FDIs after they graduated from university, which is worrying [13]. However, pharmacists could be up to date with new information about FDIs in an informal way.

In light of the rise in drug consumption and several food trends popular in recent years, such as consuming a variety of dietary supplements, restriction diets, vegetarianism, etc., we aimed to assess the level of knowledge about FDIs among Croatian pharmacists. As previous studies confirmed, improving FDI knowledge leads to improved patient care, better patient-related outcomes, a reduced number of ADEs, and reduced costs of treatment [14].

## 2. Materials and Methods

### 2.1. Study Design and Participant Recruitment

Participants included in this study were pharmacists with a license for independent work, employed in the Republic of Croatia in healthcare institutions, who agreed to participate anonymously in an online survey. To examine the knowledge of pharmacists, a cross-sectional study was conducted using an anonymous survey questionnaire during June 2022. The processed data were collected online through a Google Forms form, the link for which was forwarded to pharmacists in the Republic of Croatia with the help of the director of pharmacy institutions. This study was approved by the Ethics Committee of the Faculty of Medicine of the University of Split.

### 2.2. Data Collection and Measures

Participation was voluntary, and participants were informed that by filling out the questionnaire, they were giving their informed consent to participate. The survey questionnaire used was translated into Croatian and was taken from the research of Zawiah et al. [15]. The questionnaire is available as Supplementary Material. Although a pilot study was conducted to validate the questionnaire in the previously mentioned research, another pilot study with 10 pharmacists not later included in the study was carried out to confirm clarity and relevance to the Croatian pharmacists’ population. Prior to administering the questionnaire, drugs mentioned in the questionnaire were checked against the Annual Report on Drug Utilization in Croatia 2023 to confirm that the questions were relevant to the Croatian healthcare system [16].

The questionnaire consists of four parts and contains a total of 39 questions. The first part includes the collection of socio-demographic data such as age, gender, qualifications, place of graduation, field of work, and self-assessment of knowledge with three general questions related to FDIs (“Do you think you have enough knowledge about food-drug interactions?”, “Which of the following age groups is most susceptible to food-drug interactions?”, and “What is your main source of information about food-drug interactions?”). The second part of the questionnaire consists of 12 closed-ended questions (yes/no/do not know) that assessed the general knowledge of the pharmacists who participated in the research. Common FDIs are included in this section. Some of them could result in serious side effects (grapefruit with atorvastatin; amiodarone and some antibiotics, such as erythromycin; MAOIs with cheese and fermented foods; spironolactone with foods rich in potassium; warfarin with green vegetables; theophylline with excessive coffee and tea). Other FDIs could result in a reduction in the effectiveness of the drugs to a large extent, especially when food is consumed regularly, in large quantities, or in addition to drugs with a narrow therapeutic range (tetracycline with milk and milk products; digoxin with wheat bran; levodopa with food rich in proteins; levothyroxine and cauliflower; diazepam and caffeine). The third part included twelve closed-ended questions to assess the participants’ knowledge of the appropriate time schedule for taking medication in relation to food (half an hour before, with a meal, 2 h after a meal, and can be taken independently of a meal). The last part of the questionnaire contains 6 questions about alcohol–drug interactions.

### 2.3. Data Analysis

The total score of the questionnaire was calculated and expressed as a percentage of correct answers. Data were presented as whole numbers, percentages, and mean ranks. Data were tested for normal distribution using the Shapiro–Wilk test, which stated abnormal data distribution. The Mann–Whitney U test was used to compare test results between binary groups such as sex and age (younger than 30 and older than 30). The Kruskal–Wallis test was used to compare the total score of participant-based variables with more than 2 categories (knowledge about FDIs; source of information about FDIs). The Mann–Whitney U test with Bonferroni corrections (*p* < 0.0167) was used to compare the total score between the groups afterward. Statistical analysis was performed using IBM SPSS Statistics software (version 25). Statistical significance was set at *p* < 0.05.

## 3. Results

### 3.1. Demographic Characteristics of Participants

This study included 206 pharmacists working in Croatia. Table 1 shows the demographic characteristics of the participants. The majority of participants were female (86.4%). The median age of the participants was 29.5 years (range: 23–66)**.**

### 3.2. General FDIs Knowledge

Most of the participants (90.3%) correctly identified the elderly as the most susceptible group to FDIs. Participants had the highest score in the part of the questionnaire regarding the most common FDIs (75.00%) and the lowest score in the part regarding the appropriate time schedule for taking medication (50.00%). The median percentage of the total questionnaire score was 69.44%.

As Table 2 shows, the largest number of pharmacists were aware of the possible interaction of theophylline with coffee and tea (95.2%) and the interaction of warfarin with leafy green vegetables (94.2%). The lowest-scored question was about wheat bran diet and digoxin interaction, with only 38.9% of participants responding correctly. When asked about the timing of drug intake with respect to food, the largest percentage of participants demonstrated knowledge of omeprazole dosing (95.2%) and levothyroxine dosing (96.6%). On the other hand, the question about isotretinoin timing of intake concerning food had the smallest amount of correct answers (19.2%). Over half the pharmacists answered all questions about alcohol–drug interactions correctly, with the largest percentage of correct answers being about the paracetamol–alcohol interaction (82.7%).

### 3.3. Comparison of Questionnaire Scores Between Groups Based on Demographic Characteristics

As seen in Table 3, there is no difference in the test results between participants based on age, sex, or faculty of graduation. Also, there is no difference in total nor partial test scores between groups divided on the main source of information about FDIs. However, participants who stated they had a sufficient level of knowledge about FDIs scored significantly higher compared to those who labeled their knowledge as insufficient and those who were uncertain (128.35 vs. 102.91 vs. 89.10 denotes sufficient, insufficient, and uncertain mean ranks; *p* = 0.002). Moreover, participants with a higher valuation of their knowledge had significantly higher scores when individually compared to the “insufficient” group (*p* = 0.011) and “uncertain” group (*p* = 0.001).

## 4. Discussion

In the present study, pharmacists showed strong FDI knowledge, with 75% accuracy, and most of them identified the elderly as the most vulnerable patient group. Confidence in FDI knowledge correlated with higher scores, though the scores did not vary by age, sex, or graduation faculty. A similar study was conducted in Ethiopia where pharmacists scored 53%, which is a significantly lower score compared to the participants in this study [11]. Malaysian pharmacists scored 72.5% on the same questionnaire as our participants [13]. Another study using the same questionnaire was conducted in Jordan, where pharmacists showed similar levels of knowledge to our participants [15]. However, there are ethnic differences in pharmacokinetics that should be taken into account when comparing our results to other studies. However, it has been noted that polymorphism in enzyme genes, although very common, did not demonstrate consistent clinical relevance, with the majority of the articles reporting similarities in pharmacokinetics of different ethnic groups [17].

Multiple studies were conducted to assess FDI knowledge in the population outside pharmacists. For instance, a study of Saudi pharmacy students reported a lower level of knowledge regarding alcohol–drug interactions compared to knowledge about FDIs [18]. Biomedical students in Poland thought that it would be beneficial to learn about FDIs in a separate course as only 12% of them thought that they had a good or very good level of knowledge. Pharmacy students had the best scores among them when assessing knowledge about FDIs [19]. Although half of the nutrition students participating in the Portuguese study had a subject dedicated solely to FDIs, more than 90% of them felt that they should have been even more educated on the topic [20].

Unsurprisingly, a Saudi Arabian study that administered a similar questionnaire to the general public found even lower knowledge scores regarding FDIs [21]. A recent study in South Africa has also shown that patients had a low level of knowledge about FDIs. Half of the patients thought they were not well informed and had shown a willingness to learn more about the topic. The problematic fact is that the majority of the patients who have experienced ADEs due to FDIs did not report it [22]. It is interesting to observe the expected decrease in the level of knowledge from HCPs over students to patients and the general public. Improving overall knowledge should start at the top as obtaining an adequate level of knowledge is crucial for pharmacists and other HCPs to properly educate patients [13]. The results of our research indicate high levels of knowledge among pharmacists about FDIs, but future studies should examine how well this knowledge is translated into their daily practice and how often patients are routinely counseled on this topic.

There was no difference in knowledge of FDIs between participants who used different sources of information. The largest number of participants reported unofficial internet sources as the main source of information. This differs from previous studies, as Jordan and Palestinian pharmacists stated that university education was their main source of information, while biomedicine students in Poland identified the drug Summary of Product Characteristics (SmPC) as the primary information source [15,19,23]. Unofficial internet sources could play a crucial part in documenting FDIs as platforms where HCPs and even patients share information about FDIs could be utilized to integrate traditional sources of information with real-time patients and HCPs’ input. Moreover, a user-friendly interface based on keyword search functions, data suggestions, and FDIs classification makes them easy and quick to use while communicating with patients, as stated in the digital health policy, which encourages HCPs and patients to use technology to improve healthcare as much as possible. Drug information platforms like Drugs.com, Medscape, WebMD, and others are already part of pharmacists’ routines [24]. A potential problem with unofficial internet sources is that they are inconsistently validated, often lack a clear data source, and no one guarantees their accuracy. Recently, there has been a lot of effort put into presenting institutionalized Internet sources. The FooDrugs project is the first centralized database that used text mining to gather all available information about FDIs in the same place [25].

This study has certain limitations. The first limitation is the small number of participants considering the total number of pharmacists with authorization for independent practice in the Republic of Croatia. Also, the random sampling method was not utilized. Since participants completed an online survey without supervision, it is possible that they searched for correct answers on the Internet. This study included only pharmacists and no other HCPs or biomedicine students, which would give an even better perspective about the subject. Nevertheless, this is one of the first studies on this topic in Croatia, and even in Eastern Europe, making these results interesting not only for pharmacists but also for educators in the field of FDIs. Furthermore, another limitation is the survey questionnaire, which was developed based on the previously published literature and does not take into account the most commonly used medications in Croatia. The questionnaire consisted of yes/no questions, which gives the possibility of a correct answer by chance. A better assessment of knowledge could be collected with open-ended questions. Future research should include annual drug consumption data when developing tools for assessing knowledge of FDIs.

## 5. Conclusions

Although the level of knowledge among Croatian pharmacists shown in this study was relatively high compared to similar studies, certain shortcomings were present. The educational strategy of FDIs should take into account the results from this and future studies about the topic to deliver precise and understandable information to HCPs, students, and patients. Increased knowledge on this matter would ultimately lead to the improved management of FDIs and improved patient outcomes.

## Figures and Tables

**Table 1:** Demographic characteristics of participants.

		N (%)
**Sex**	Female	178 (86.4)
	Male	28 (13.6)
Faculty of graduation	USSM ^1^	71 (34.5)
	FBF ^2^	135 (65.5)
Chronic medication use	No	164 (79.6)
	Yes	42 (20.4)
Self-assessed knowledge about FDIs	Sufficient	51 (24.8)
	Insufficient	85 (41.3)
	Uncertain	70 (34.0)
The main source of information about FDIs	Official sources	68 (33.0)
	Unofficial internet sources	83 (40.3)
	Formal education	33 (16.0)
	Experience	5 (2.4)
		Median (Range)
Age		29.5 (23–66)

^1^ University of Split School of Medicine, ^2^ University of Zagreb Faculty of Pharmacy and Biochemistry.

**Table 2:** Number (percentage) of correct answers on the 3 best answered questions and 3 worst answered questions from the first part of the questionnaire.

Question	N (%)
Patients taking theophylline should avoid excessive coffee and tea	198 (95.2)
Patients can eat more leafy green vegetables with warfarin	196 (94.2)
Milk affects the efficacy of tetracycline	192 (92.3)
Protein-rich foods affect the efficacy of levodopa	113 (54.3)
Cauliflower consumption affects the efficacy of levothyroxine	91 (43.8)
Wheat bran diet affects the efficacy of digoxin	81 (38.9)

**Table 3:** Comparison between partial and total test scores between the groups.

		2nd Part Score		3rd Part Score		4th Part Score		Total Score	
N = 206		Mean Rank	*p* *	Mean Rank	*p* *	Mean Rank	*p* *	Mean Rank	*p* *
Age	30 or younger	103.29	0.751	102.81	0.655	99.35	0.172	99.98	0.241
	Over 30	105.92		106.47		110.51		109.78	
Sex	Female	104.36	0.929	105.63	0.485	106.49	0.214	105.76	0.442
	Male	105.43		97.27		91.68		96.38	
Faculty of graduation	MEFST	113.66	0.106	105.00	0.929	111.17	0.232	111.44	0.225
	FBF	99.65		104.24		100.97		100.83	
Self-assessed knowledge about FDIs	Sufficient	127.04	0.000 **	110.76	0.680	126.49	0.006 **	128.35	0.002 **
	Insufficient	107.21		102.08		100.68		102.91	
	Uncertain	84.71		102.94		93.22		89.10 vs	
The main source of information about FDIs	Official sources	99.37	0.812	100.62	0.598	98.90	0.632	100.43	0.834
	Unofficial INTERNET sources	91.61		96.59		96.24		94.13	
	Formal education	98.77		88.89		86.42		90.77	
	Experience	98.29		77.64		110.07		99.79	

* *p*-values calculated from the Mann–Whitney U test for binary comparisons and Kruskal–Wallis test for multi-category comparisons. ** *p* < 0.005.

## Data Availability

Data are available on reasonable request to the corresponding author.

## Supplementary Materials

The following supporting information can be downloaded at: https://www.mdpi.com/article/10.3390/pharmacy12060172/s1, File: The questionnaire used in the study.
