# Supplementary material for: Evaluating Pharmacists’ Knowledge of Food–Drug Interactions in Croatia: Identifying Gaps and Opportunities
Source: Pharmacy (Basel). 2024 Nov 21;12(6):172. doi: 10.3390/pharmacy12060172 (PMC11587475; doi:10.3390/pharmacy12060172)
Supplement: Supplementary file 1 [file pharmacy-12-00172-s001.zip › pharmacy-3268857-supplementary.pdf]

Food-drug interactions: Knowledge among pharmacists in Croatia

(Questionnaire)

Part (1). Demographics and general questions:

- Age: ....

- Gender:

☐ Male ☐ Female

- Education level:

☐ Bachelor of pharmacy ☐ Doctor of pharmacy ☐ Master

- Name of the university (For your last degree): ....

- Area of work now:

☐ Community pharmacy ☐ Hospital pharmacy

- Number of experience years: ....

- Employment status:
☐ Employee ☐ Owner

- Do you think you have enough information about food-drugs interactions?
☐ Yes ☐ No ☐ Not sure

- Which of the following age groups is most susceptible to food-drug interactions?
☐ Children ☐ Adults ☐ Elderly

- **What is the main source for your knowledge of food-drug interactions?**

…………………………….

Part (2). Drugs-Food Interactions knowledge

1. Can amiodarone be taken with grapefruit?
☐ Yes ☐ No ☐ I don’t know

2. Can atorvastatin be taken with grapefruit?
☐ Yes ☐ No ☐ I don’t know

3. Does cauliflower consumption affect the efficacy of levothyroxine?
☐ Yes ☐ No ☐ I don’t know

4. Does caffeine consumption affect the efficacy of diazepam?
☐ Yes ☐ No ☐ I don’t know

5. Patients can eat more leafy green vegetables with Coumadin (warfarin):
☐ Yes ☐ No ☐ I don’t know

6. Patient taking theophylline should avoid excessive coffee and tea:
☐ Yes ☐ No ☐ I don’t know

7. Does milk affect the efficacy of tetracycline?
☐ Yes ☐ No ☐ I don’t know

8. Patients taking monoamine oxidase inhibitors (MAOIs) should avoid eating aged cheeses:
☐ Yes ☐ No ☐ I don’t know

9. Does wheat bran diet affect the efficacy of digoxin?
☐ Yes ☐ No ☐ I don’t know

10. Does protein-rich foods affect the efficacy of levodopa?
☐ Yes ☐ No ☐ I don’t know

11. Grapefruit juice can be safely consumed with all antibiotics:
☐ Yes ☐ No ☐ I don’t know

12. Patients should avoid taking spironolactone with food rich in potassium?
☐ Yes ☐ No ☐ I don’t know

Part (3). Knowledge about timing of drug intake with respect to food:

Please choose the best time to take each medication with respect to food

	Medication	Before meal with 1/2 hour	With meal	Two hours after meal	Can be taken without regard to food
13.	Carbamazepine				
14.	Methotrexate				
15.	Isotretinoin				
16.	Omeprazole				
17.	Glipizide				
18.	NSAIDs				
19.	Levothyroxine				
20.	Griseofulvin				
21.	Metformin				
22.	Calcium carbonate supplement				
23.	Erythromycin stearate				
24.	Propranolol				

Part (4). Knowledge about Drugs-alcohol interactions:

For each medication, please choose whether there is drug-alcohol interaction or not (“Yes” means **there is** drug-alcohol interaction, “No” means **there is no** drug-alcohol interaction, “I don’t know” means you do not know the answer)

	Medication	Yes	NO	I don’t know
25.	Antihistamine			
26.	Paracetamol			
27.	Metformin			
28.	Isoniazid			
29.	Warfarin			
30.	Methotrexate			

Thank you
